# Does hyperoxia enhance susceptibility to secondary pulmonary infection in the ICU?

**DOI:** 10.1186/s13054-016-1427-x

**Published:** 2016-08-16

**Authors:** Benedikt Nußbaum, Peter Radermacher, Pierre Asfar, Clair Hartmann

**Affiliations:** 1 Institut für Anästhesiologische Pathophysiologie und Verfahrensentwicklung, Universitätsklinikum, Helmholtzstrasse 8/1, Ulm, Germany; 2 Klinik für Anästhesiologie, Abteilung Klinische Anästhesiologie, Universitätsklinikum, Albert-Einstein-Allee 23, Ulm, Germany; 3 Département de Réanimation Médicale et de Médecine Hyperbare, Centre Hospitalier Universitaire, 4 rue Larrey, Angers, France; 4 Laboratoire de Biologie Neurovasculaire et Mitochondriale Intégrée, CNRS UMR 6214 - INSERM U1083, Université Angers, PRES L’UNAM, Angers, 49933 Cedex 9 France

**Keywords:** Hyperoxia, Mechanical ventilation, Antibiotic, Ventilator-associated pneumonia, Phagocytosis, Catecholamine, Sedation

## Abstract

Hyperoxia is common practice in the acute management of circulatory shock, and observational studies report that it is present in more than 50 % of mechanically ventilated patients during the first 24 h after intensive care unit (ICU) admission. On the other hand, “oxygen toxicity” due to the increased formation of reactive oxygen species limits its use due to serious deleterious side effects. However, formation of reactive oxygen species to boost bacterial killing is one of the body’s anti-microbial auto-defense mechanisms and, hence, O_2_ has been referred to as an antibiotic. Consequently, hyperoxia during the peri-operative period has been advocated for surgical patients in order to reduce surgical site infection. However, there is ample evidence that long-term exposure to hyperoxia impaired bacterial phagocytosis and thereby aggravated both bacterial burden and dissemination. Moreover, a recent retrospective study identified the number of days with hyperoxia, defined as a PaO_2_ > 120 mmHg only, as an independent risk factor of ventilator-associated pneumonia in patients needing mechanical ventilation for more than 48 h. Since so far the optimal oxygenation target is unknown for ICU patients, “conservative” O_2_ therapy represents the treatment of choice to avoid exposure to both hypoxemia and excess hyperoxemia.

## Background

Despite recent evidence that “conservative” oxygenation targets are safe [1, 2], hyperoxia is frequently present in the intensive care unit (ICU) [3, 4]. O_2_ has friend-and-foe properties because it is vital for ATP production and toxic due to its oxidant effects [5]. On the one hand, “…administration of oxygen should be started immediately to increase O_2_ delivery…” during the management of circulatory shock in order to counteract the pathognomonic “…imbalance between O_2_ supply and requirements…” [6]. On the other hand, oxygen toxicity arises from the formation of reactive oxygen species (ROS), in particular during hypoxia/re-oxygenation, e.g., during shock resuscitation. ROS themselves share the janus-headed character of O_2_: they are vital signaling molecules and their increased release to boost bacterial killing is one of the body’s anti-microbial auto-defense mechanisms [7]. ROS formation is directly related to the PO_2_ [5] and, hence, O_2_ was already described as an antibiotic three decades ago [5]. Again, there is contrasting experimental evidence: long-term (48–96 h) exposure to PaO_2_ values >350–400 mmHg aggravated the pulmonary bacterial burden and the bacteria dissemination [8] due to impaired bacterial phagocytosis and endotoxin-induced cytokine release. Interestingly, ROS scavengers dose-dependently restored the bactericidal capacity.

Hyperoxia during the peri-operative period was reported to reduce post-operative wound infection. Pure O_2_ ventilation attenuated the anesthesia- and surgery-induced impairment of the phagocytic and microbicidal capacity of alveolar macrophages in healthy patients undergoing prolonged (>6 up to 10 h) elective surgery [9]. The most recent meta-analysis (>8000 patients in 17 randomized control trials (RCTs)) concluded that hyperoxia significantly decreases the risk of surgical site infection during colorectal surgery [10]. Nevertheless, this approach remains highly controversial due to the “moderate evidence” [10] and the deleterious long-term complications reported in patients with cancer [11] and/or cardiovascular disease [12]. Moreover, the question remains unanswered whether hyperoxia affects host defense in mechanically ventilated ICU patients in general: using a multivariate analysis of retrospective data on patients needing mechanical ventilation for >48 h, Six et al. recently identified the number of days with hyperoxia as an independent risk factor of ventilator-associated pneumonia (VAP) [13].

### Hyperoxia, lung function, and O_2_ transport

Hyperoxia impairs pulmonary gas exchange due to inhibition of hypoxic pulmonary vasoconstriction and adsorption atelectasis [5]. Moreover, under healthy conditions blood O_2_ content only modestly increases upon switching from air to pure O_2_ breathing due to the near-complete arterial hemoglobin O_2_ saturation (SaO_2_) at PaO_2_ = 90–100 mmHg [5]. Hence, the lower the hemoglobin content, the more pronounced the effect of hyperoxia on blood O_2_ content, such that hyperoxia may be helpful during hemorrhage [5]. However, any hyperoxia-related rise in blood O_2_ content may at least in part be counterbalanced by a hyperoxia-induced fall in cardiac output resulting from decreased heart rate and increased systemic vascular resistance, the latter being particularly pronounced in the cerebral and coronary vasculature [5].

The acute hyperoxia-induced impairment of gas exchange must be discriminated from pulmonary O_2_ toxicity, which presents as pulmonary inflammation and, ultimately, hemorrhagic pulmonary edema [5]. This effect is well-established after long-term hyperoxic exposures and/or when combined with injurious ventilation. The only data in mechanically ventilated patients originate from hyperoxia over 14 h to 30 days and, given the publication year (1972), lung-protective ventilation most likely was not used [5].

### Hyperoxia and secondary pulmonary infection

The study by Six et al. [13] clearly suggests an association between hyperoxia and VAP. Of note, hyperoxia was defined as a PaO_2_ > 120 mmHg, in other words, a PaO_2_ that is commonly observed in ICU patients [3, 4]. Moreover, the authors showed not only that the numbers of days with hyperoxia, but also “hyperoxemia at ICU admission”, in other words, deliberate, iatrogenic pre-ICU hyperoxia, was an independent risk factor of VAP. Clearly, patients with VAP were older, sicker, and, in particular, more frequently in shock prior to ICU admission. First, shock, as the dysbalance between tissue O_2_ supply and demand leads to tissue hypoxia, which in turn triggers hyper-inflammation that is aimed to clear pathogens [14]. However, when “too pronounced and/or sustained”, tissue hypoxia may cause anti-inflammation, thereby rendering patients more susceptible to secondary infection [14]. Second, shock is defined as arterial hypotension and/or the need for vasopressor use. Hence, in the study by Six et al., patients with VAP most likely received catecholamines more frequently, for a longer period, and/or at higher doses. Exogenous catecholamines can impair both innate and adaptive immunity and thereby increase bacterial growth and virulence. Despite these potential limits and confounding factors, the study by Six et al. raises an important question with respect to the use of hyperoxia, i.e., whether there is an optimal PaO_2_ target in the ICU. So far, only retrospective data are available yielding a U-shaped relationship between mortality and arterial PO_2_, with a nadir at PaO_2_ values of 110–150 mmHg [3] and 150–200 mmHg [15]. Mortality sharply increased at PaO_2_ < 65 mmHg and >225 mmHg [3]. However, another study found that even PaO_2_ > 300 mmHg did not affect outcome, while confirming the harmful impact of PaO_2_ < 65 mmHg [4]. Consequently, despite a strong signal suggesting an association between hyperoxia and poor hospital outcome, the most recent meta-analyses on hyperoxia [16, 17] are inconclusive due to the high data heterogeneity.

## Conclusions

Hyperoxia is common practice during shock management based on experimental evidence that correcting oxygen debt is crucial for survival. On the other hand, oxygen toxicity limits its use, especially during hypoxia/re-oxygenation and/or long-term administration (i.e., >12–24 h). The data of the RCT “Optimal Oxygenation in the Intensive Care Unit (O2-ICU)” (NCT02321072) comparing PaO_2_ targets of 120 versus 75 mmHg in ICU patients and the detailed results of the preliminary terminated 2 × 2-factorial RCT “Hyperoxia and Hypertonic Saline in Septic Shock (Hyper2S)” (NCT01722422), simultaneously comparing target SaO_2_ 88–95 % versus pure O_2_ ventilation during the first 24 h and isotonic versus hypertonic saline, are therefore eagerly awaited. The results may allow finding criteria for “…a personalized O_2_ target…in critically ill patients” [14]. Until then, conservative O_2_ therapy [1, 2] should be the treatment of choice to avoid both hypoxemia and excess hyperoxia.

## Abbreviations

ICU, intensive care unit; RCT, randomized controlled trial; ROS, reactive oxygen species; SaO_2_, arterial hemoglobin oxygen saturation; VAP, ventilator-associated pneumonia
